# Amelanotic Malignant Mucosal Melanoma of the Nasal Cavity: Case Report and Literature Review

**DOI:** 10.7759/cureus.22442

**Published:** 2022-02-21

**Authors:** Ilias Tahiri, Othman El Houari, Amal Hajjij, Mohammed Zalagh, Fouad Benariba

**Affiliations:** 1 Otolaryngology - Head and Neck Surgery, Faculty of Medicine, Mohammed VI University of Health Sciences (UM6SS), Casablanca, MAR

**Keywords:** pet ct scan, hemi-maxillectomy, melanocytic markers, cancer immunotherapy, mucosal malignant melanoma

## Abstract

Sinonasal malignant melanoma is a rare but aggressive tumor of the head and neck area. It has a poor prognosis. Common symptoms are nasal obstruction, epistaxis, or purulent rhinorrhea. Diagnosis relies on histopathology with immunohistochemistry (IHC) studies. Surgery is the essential treatment, most often supplemented by radiotherapy or immunotherapy. A 63-year-old female patient, with a history of right dacryocystorhinostomy and Parkinson's disease, consulted for symptoms of right nasal obstruction with increasing intensity accompanied by two episodes of mild unilateral epistaxis. Rigid optic examination showed a white-pinkish right obstructive supra-centimetric endonasal tumor. CT revealed an extensive tissue process of the right nasal cavity invading the maxillary sinus, the inferior and middle conchas. A biopsy of the lesion was conducted under local anesthesia. The immunohistochemical study has shown undifferentiated tumor with positive antibody anti PS100 and anti-melan A evoking malignant sinonasal melanoma. The patient underwent two surgeries for maxillectomies as she presented a first local recurrence. She was started on adjuvant radiotherapy. At one year of follow-up, she does not present any local or general signs of disease. Sinonasal melanoma is a particular entity of head and neck mucosal melanomas. The highest incidence is described to be in the seventh and eighth decades of life with no sex difference. IHC profiling of different melanoma subtypes showed the importance of alterations in the KIT gene, this genetic data may constitute a therapeutic target. After surgery, the important local recurrence rates and regional failure justify adjuvant radiotherapy also for resections in free margins. Most authors consider that prophylactic neck dissection is not necessary. Preoperative imaging features (CT scan) are characteristic and helpful for diagnosis. IHC is essential, has a high sensitivity for differentiating achromic melanomas from other neoplasms. Sinonasal achromic melanoma is a very uncommon tumor, invasive, and frequently associated with distant metastasis. Paraclinic examinations are essential for staging and guiding therapeutic management. Immunotherapy is a promising ground of research as it comes to metastatic and advanced disease.

## Introduction

Malignant melanoma of the head and neck (HNMM) is a rare and aggressive tumor with a poor prognosis (about 30% at five years) [1]. HNMM can involve all the aerodigestive tract, including the oral cavity, the pharyngo-larynx, the nasal fossa, and the paranasal sinuses. It is a malignant tumor proliferating from melanocytes of neuroectodermal origin with or without melanin pigment.

Mucosal melanomas differ from cutaneous melanomas by their pathobiology and clinical presentation, as the mucosal form of melanoma is not correlated with solar exposure and also exhibits other cytogenetic alterations (i.e., KIT gene mutations) [2]. Around 50% of all mucosal melanomas are in the head and neck region. The nasal cavity is a rare localization for primary malignant melanoma and represents less than 1.5% of all head and neck tumors [3]. The unpigmented variety known as achromic melanoma is exceptional.

It is known that most HNMMs are diagnosed at a late stage (1) because the tumor process evolves insidiously without clinical symptoms. Common symptoms are represented by nasal obstruction, epistaxis or rhinorrhea, and less frequent pain. Mostly, at the time of diagnosis, lesions have developed, and lymph nodes or metastases are present (up to 10%-30%) [4]. Histologically, the presence of melanin on the epithelium originating from neoplastic melanocytes is the hallmark feature of this lesion. The amelanotic variant that does not produce melanin, constitutes a diagnostic challenge for pathologists, requiring immunohistochemistry (IHC) studies [5].

The treatment is discussed and determined in a multidisciplinary consultation meeting. It is mainly surgical, most often supplemented by radiotherapy or immunotherapy. On the other hand, surgical treatment for advanced mucosal melanomas can be deleterious because of functional prognosis, cosmetic considerations, and non-oncological resection leading to high local and general recurrence rates. We report the case of a rare presentation of an achromic melanoma of the sinonasal cavity, with a review of the previously reported cases of mucosal melanomas of the head and neck area.

## Case presentation

We report a case of a 63-year-old female patient with a history of osteoarticular tuberculosis declared cured in 2010, Parkinson's disease under medical treatment for one year, and a right dacryocystorhinostomy in 2015. She consulted in March 2021 at the Otorhinolaryngology Department of Cheikh Khalifa Hospital in Casablanca for rhinological symptoms evolving for 02 months. The patient reports symptoms of intermittent and then permanent right nasal obstruction of increasing intensity accompanied by two episodes of mild unilateral epistaxis in a context of altered physical and mental functions.

**Figure 1:**
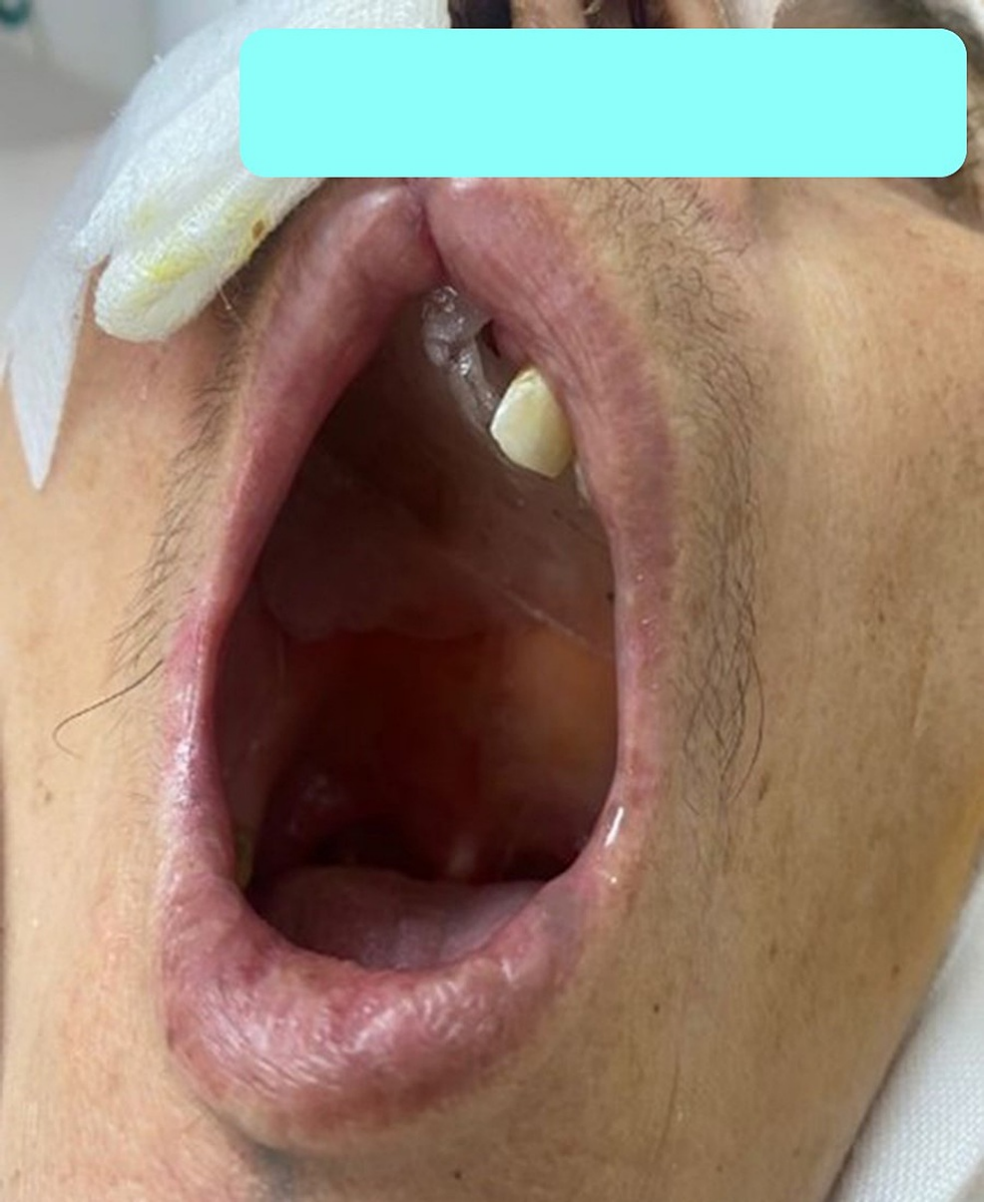
Infiltration of the ulcerative tumor invading the hard palate.

The rigid optic examination showed a white-pinkish right obstructive supra-centimetric endonasal tumor process encroaching onto the nasal septum bleeding when in contact. The right hard palate was infiltrated (Figure 1). There was no palpable cervical lymphadenopathy (submandibular and superior jugulo-carotid areas). The rest of the somatic examination including oral cavity, pharyngolarynx (direct laryngoscopy) skin, and appendages showed no particularities. A biopsy-excision with immunohistochemical study has diagnosed undifferentiated tumor with IHC antibody anti PS100 and anti-melan A partially positive evoking malignant melanoma.

**Figure 2:**
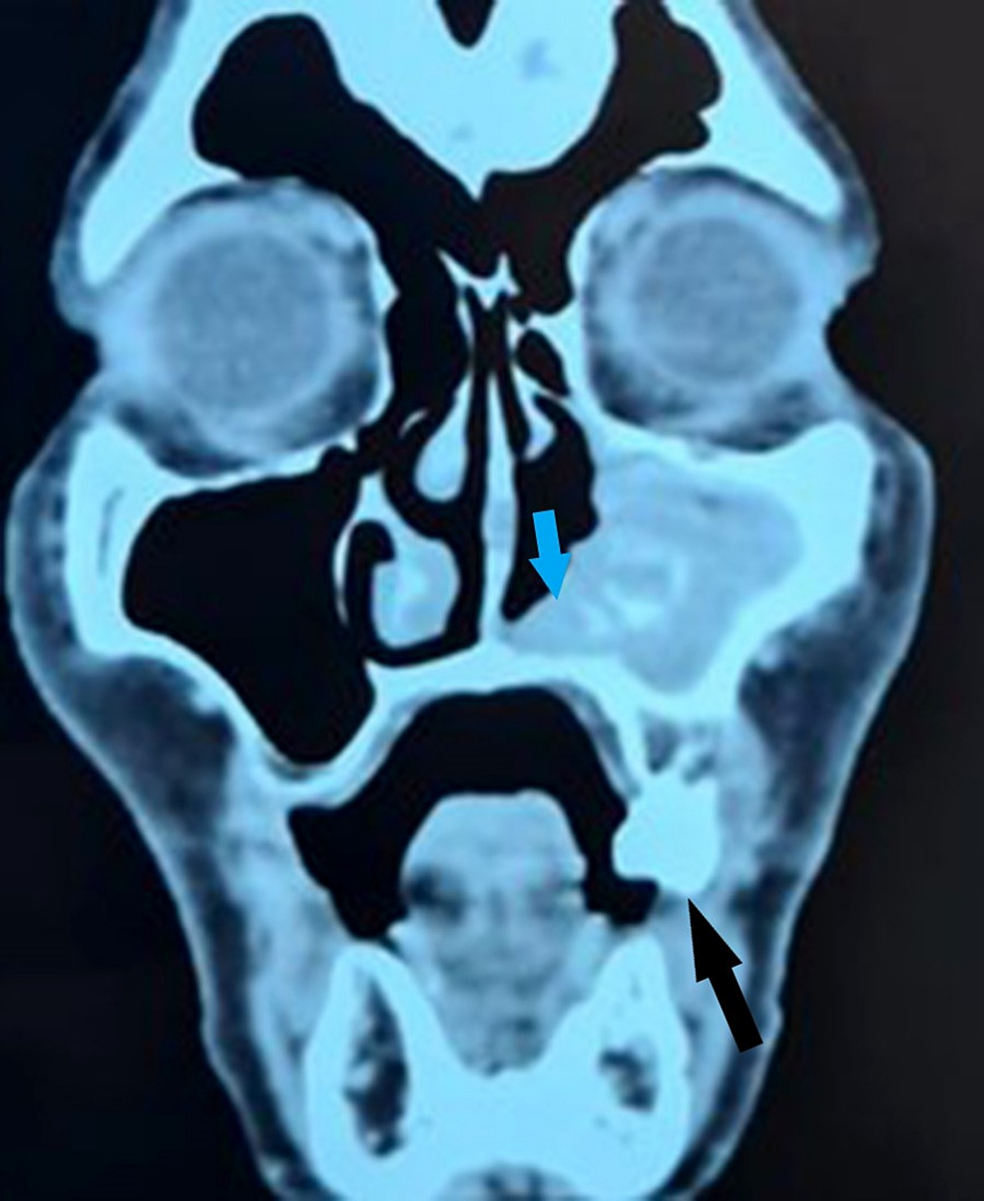
Scannographic presentation of the right sinonasal mucosal melanoma (hypodense structure invading the inferior concha and the floor of the maxillary sinus). Black arrow, tumor invasion to the floor of the right maxillary sinus; blue arrow, destruction of the right inferior concha

The CT examination of the facial mass revealed an extensive tissue process of the right nasal cavity invading the maxillary sinus, the inferior and middle conchas with respect to the ethmoid sinus, and the bony structures (Figure 2). Cervico-thoracic CT showed mediastinal lymphadenopathy. Abdominal ultrasound has not revealed any secondary lesions. The positron emission tomography (PET) scan examination found a hypermetabolic process of the right nasal cavity pushing back the nasal septum, in contact with the lateral wall of the ipsilateral maxillary sinus with associated bone lysis. Moreover, low-grade hypermetabolic para-tracheal, right lobar, interlobar, and bilateral hilar lymphadenopathies were described (inflammatory adenopathies).

The therapeutic protocol decided by the multidisciplinary consultation meeting was to start with embolization of the right sphenopalatine and lingual arteries. The aim was to avoid revision surgery as much as possible due to the aggressive and recurrent nature of the disease. A right medial maxillectomy was performed to allow for complete excision of the tumor. The final pathological examination confirmed the diagnosis of malignant melanoma. The patient received three postoperative radiotherapy sessions of 45 Gy. The patient was not able to complete her chemotherapy sessions due to cardiac arrhythmias. In the follow-up, she came to consult for a right maxillary purulent swelling five months after surgery. The diagnosis of loco-regional recurrence with the invasion of the anterior, posterior, lateral wall, and floor of the right maxillary sinus and the right nasal cavity was posed.

**Figure 3:**
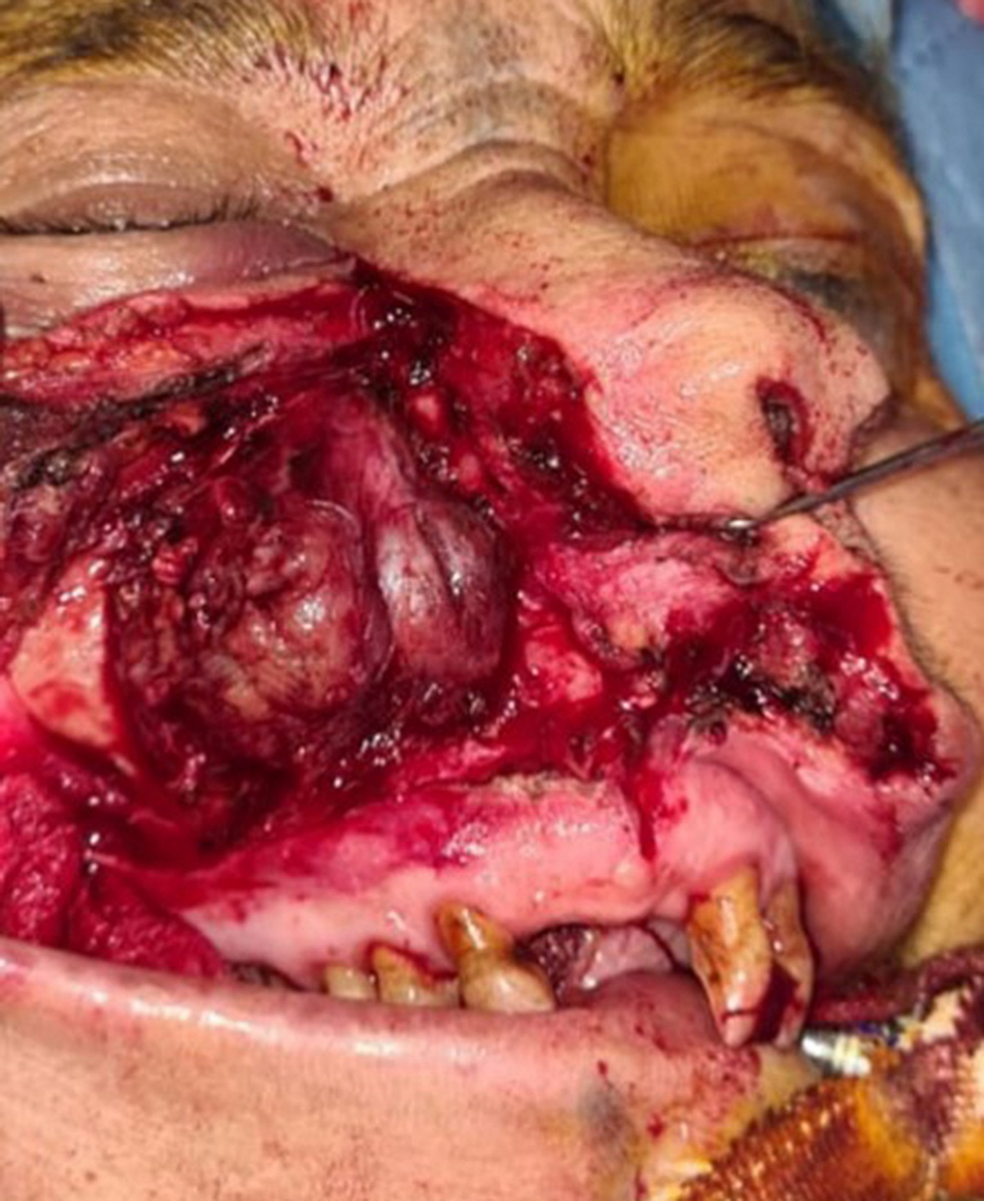
Preoperative aspect after resection of the anterior wall of the maxillary sinus.

**Figure 4:**
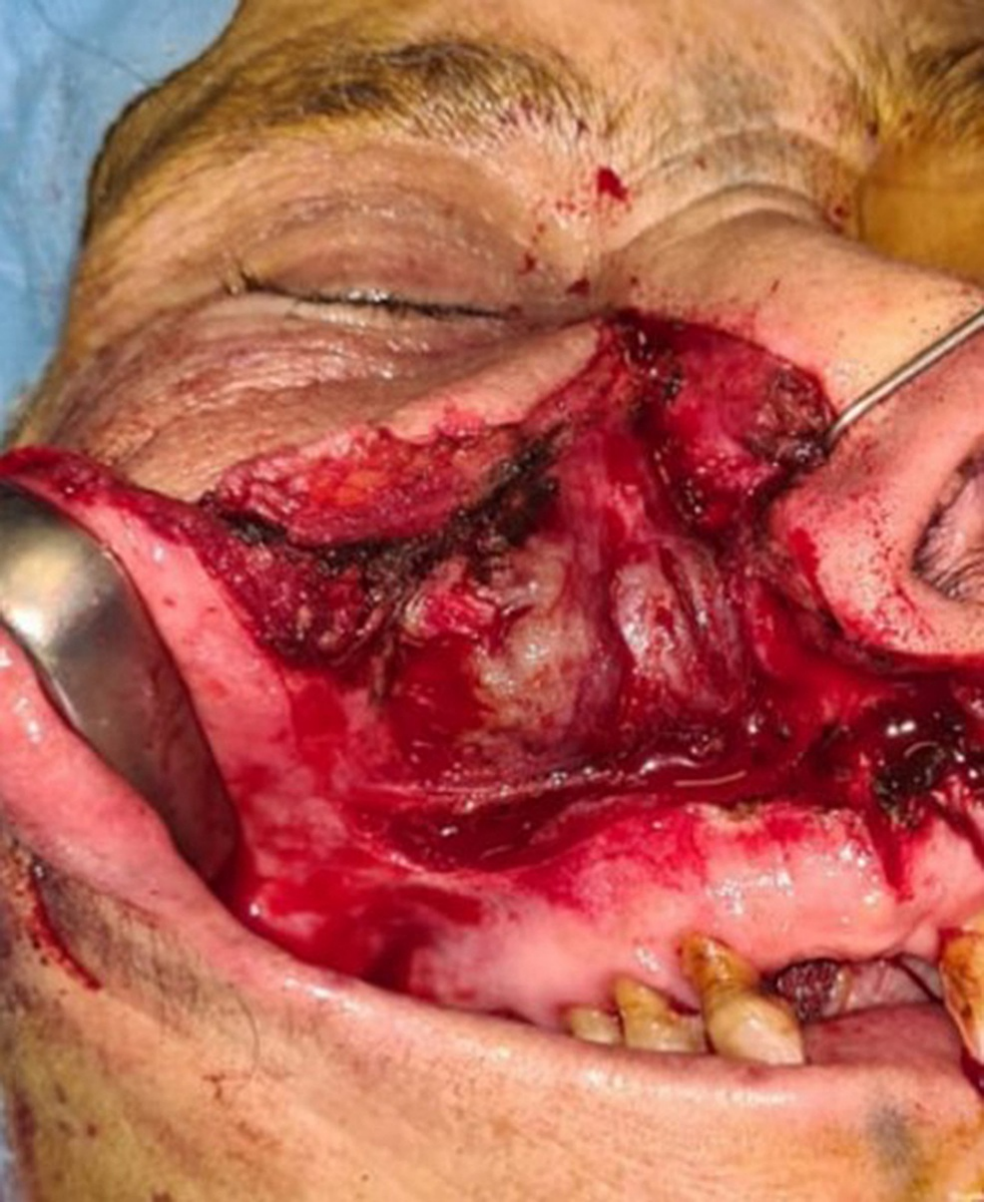
Postoperative aspect after complete excision of the lesion.

A second surgical intervention was performed by a wide right para-lateral nasal approach comprising a right subtotal maxillectomy with preservation of the orbital floor and placing of a palatal prosthesis (Figures 3-4). No neck dissection was performed as the patient was clinically and radiologically staged N0. The prosthesis was well tolerated by the patient who recovered and was started on a normal diet one month after surgery. The patient was started on adjuvant radiotherapy (45 Gy). One-year post-operative, the patient came for follow-up, with no signs of epistaxis, anosmia, or nasal obstruction. Nasofibroscopy and control CT scan showed no local recurrence.

## Discussion

Sinonasal melanoma is a particular entity of head and neck mucosal melanomas. It is a rare and aggressive lesion, with an incidence of 0.2-1 case per million [6]. Recent studies of genetic and immunohistochemical profiling of different melanoma subtypes, including HNMM and other mucosal melanomas showed the potential importance of alterations in the KIT gene and the NRAS gene in mucosal melanoma [7]. The estimated frequencies of mutations in HNMM differ between studies as follows; KIT: 15%-40%, NRAS: 40%-50%, and BRAF: 0-3%. Given the probable pathogenic role of the KIT gene in mucosal melanomas, detection of KIT aberrations may present potential diagnostic value, and this genetic data may constitute a therapeutic target [8]. The frequency of NRAS mutations in HNMM (~16%) is similar to that observed in cutaneous melanoma. Nevertheless, the types of NRAS alterations in HNMM vary from the types that prevail in skin melanoma. This model of NRAS mutations in mucosal melanoma suggests another etiology, different from UV radiation [9].

Patients with HNMM present a poor overall survival outcome with five-year overall rates from 20% to 50%. For most authors, this is attributed mainly to concomitant metastasis at the time of diagnosis [10]. However, there are few studies on regional metastasis in HNMM patients, and available data do not precise if this criterion is a major prognostic factor in patients with mucosal melanoma of the head and neck area [11]. The important local recurrence rates may be a consequence of the multifocal nature of the disease and less probably due to an infra-clinical lymphatic spread of mucosal melanoma. These factors underline the major importance of radical wide local excision also for early clinical stages of the disease. Recent searches have found that although no more than 10%-15% of HNMM patients have distant metastasis at the time of initial presentation, the distant failure rate is as high as 83% [12]. These observations indicate that early hematogenous progression is a significant challenge for treating HNMM, and systemic evaluation after curative resection is essential for survival outcome.

Sinonasal melanoma has been described more frequently in areas where skin melanomas are less common, which may be caused by factors associated with the disease, as aforementioned genetic features [13]. Although our case report is about a female patient, the results of research studies have shown a male predominance or no sex difference [14]. Regarding age, the highest incidence was described to be in the seventh and eighth decades of life, which correlates to our case study. Time until diagnosis documented at 6.3 months in the previous literature [14] was relatively late, certainly related to the asymptomatic evolution of the tumor in many cases.

Concerning the site of the lesion, the nasal cavity is the most affected, followed by the maxillary, ethmoid sinus, and other paranasal sinuses [15]. The main symptoms usually presented by patients with HNMM are usually epistaxis, nasal obstruction, and blurry vision. In our case, there were no visual disturbances as the tumor has not expanded enough to provoke neural invasion. Regarding lymph node metastasis, our patient has not presented any lymphadenopathy at clinical examination. Some researches have reported a low incidence of nodal disease (5.15%), while others have described a much higher rate (30%) [16]. The submandibular, jugulodigastric lymph nodes are most concerned since they are involved in the lymphatic drainage of the nose. Early diagnosis of cervical metastatic nodes could help patients for earlier treatment with surgery or adjuvant radiotherapy. It could potentially improve prognosis and the overall survival rate reducing distant metastasis.

Preoperative imaging features are characteristic and helpful for diagnosis. CT is the most common examination modality, the lesions presenting as heterogeneous hypodense images [15]. Bony erosion or destruction can occur in the posterior part of maxillary sinus, orbital floor, and skull base. MRI is less frequently used and the tumor is identified as a hypointense or hyperintense image, with better evaluation of soft structures as fat tissue of the cheek and skin [14].

The sinonasal melanoma is a very uncommon tumor, whereas the amelanotic form tends to be exceptional. On microscopic analysis, the melanotic subtype represents 86.8% of the cases and the amelanotic only 13.2%. For malignant melanoma, most tumor cells are pigmented and thus the diagnosis of melanin-rich melanoma is obvious. On the contrary, the amelanotic presentation is a challenge for histopathologists [13]. In melanoma, tumor cells are organized in sheets with oval and round nuclei with voluminous nucleoli. A similar histological structure can be recognized in many other sinonasal cancers (undifferentiated, small round cell tumors). IHC is essential for diagnostic purposes. IHC markers; S-100 and HMB-45 have high sensitivity for differentiating melanomas from other neoplasms [13].

To provide the best chance for a long-term cure, large surgical excision with free margins is the cornerstone for treating any HNMM. The size and location of the lesion influence the surgery choice (type and extent) for oncological resection. This objective remains challenging and is not uncommonly impossible. Proximity of the tumor to vital structures (such as the internal carotid artery for instance) and in some cases locally advanced disease (such as in orbital or extended dural invasions) at presentation has to be considered in the preoperative assessment. It remains unclear whether radical surgery if causing significant esthetic and functional impairments are justified. In all cases, aggressive adjuvant therapy must be used for locally advanced disease and large tumors without consideration of any margin status. Surgery as a single-mode therapy for HNMM patients is exclusively used for localized tumors [16].

The HNMM patients have a moderate rate of clinical lymphadenopathies at initial presentation (10%-30%) [5]. Neck dissection is realized for clinically positive nodes (areas I, II, III of the neck). Most authors consider that prophylactic neck dissection is not necessary for HNMM [17]. Retrospective studies compared the outcomes and overall survival of mucosal melanoma patients who have benefited from surgery alone and those who underwent surgery and adjuvant radiotherapy (RT) [18]. Postoperative RT had a positive effect for improving locoregional control but no data had demonstrated an overall survival benefit (knowing that some patients with localized tumors do not go under RT constituting a bias for the results) [18].

Several studies suggested that definitive RT did not reach as significant local control of the tumor extent as surgery [19]. Doses higher than 50 Gy have been proposed for locoregional control in adjuvant RT. Nevertheless, retrospective data of mucosal melanoma have not pointed out any clear correlation between a particular dose and survival rates [19].

The frequency of KIT gene alterations in mucosal melanomas suggests a potential utility for selective KIT inhibitors in the treatment of these tumors. For patients presenting advanced disease and c-KIT mutations, immunotherapy (tyrosine kinase inhibitor imatinib) had a disease response rate of 54% and an overall control rate of 77% [20]. The overall survival outcome of patients receiving ipilimumab, a monoclonal antibody (10.1 months) was significantly better than that of patients who were treated by the standard of care (6.4 months; p = 0.003). However, immunotherapy is a very recent therapeutic modality to treat patients suffering from mucosal melanoma, and more research has to be done to validate this kind of therapy [20].

## Conclusions

Sinonasal achromic melanoma is a very rare tumor, potentially aggressive. The diagnosis of this pathology relies on clinical examination, CT scan, and IHC after biopsy of the lesion. Extension assessment is essential for staging and guiding therapeutic management. Surgery is the cornerstone for localized tumors and radiotherapy is indicated even for radical resections in free margins. Immunotherapy is a promising ground of research as it comes to metastatic and advanced disease.
